# The Study of Estimation of Landfill Capacity through Dynamic System Approach

**DOI:** 10.1155/2022/1068111

**Published:** 2022-07-19

**Authors:** Lina Warlina, Sri Listyarini

**Affiliations:** Universitas Terbuka, Faculty of Science and Technology, Pondok Cabe, Tangerang, Selatan 15418, Indonesia

## Abstract

In line with the increase in population, the production of waste will also grow every year. Unfortunately, the land used for final waste disposal (landfill) is extremely limited, especially in developing countries. *This study aimed to predict landfill capacity* in accommodating waste from the community *based on daily waste input using a dynamic system* and to provide alternative policies on landfill waste management. The analyzed data consisted of primary and secondary data, whereas the simulations applied system dynamic approach using Vensim software. The simulation results indicate that the waste production will reach 36,861,653 tons in 2030 if nothing is done. Assuming that all waste from the surrounding communities is accumulated in one landfill, *Bantargebang landfill* can only accommodate until 2022. To be able to use *Bantargebang landfill* up to 2030, the waste production must be reduced by 50% for organic waste and 50% for inorganic waste. From the analysis, it is proven that composting reduces a greater amount of waste than other waste reduction methods, namely recycling or reusing, by the same percentage. Waste sorting is therefore highly recommended to be done by the community in every household as a basis to facilitate further handling. The community can play an active role in reducing waste, e.g., by composting organic waste and recycling or reusing inorganic waste. Furthermore, regulations should be made that can give punishment to households that do not carry out segregation. Reliable infrastructure for waste management needs to be facilitated, and counseling/training/outreach on waste sorting to the community must also be provided at the district level.

## 1. Introduction

The rapid population growth can have an impact on the waste produced [1]. The high volume of waste produced by the industrial and household sectors becomes a problem commonly found in big cities. This problem is incredibly complex and difficult to handle. Lack of land used for final waste disposal (landfill) is another important issue to address, especially in developing countries [2]. The phenomenon of overcapacity at landfills happens in numerous countries worldwide, including Malaysia [3], India [4], Australia [5], Oregon [6], China [7], and Indonesia [8].

While there are many existing landfills all over Indonesia, Bantargebang landfill is possibly the most famous one due to its vast area. Being the biggest landfill, Bantargebang landfill also faces the same condition as the other landfills in Indonesia, that is, overcapacity [8]. This situation must be avoided as it will have negative impacts on the environment. If the amount of waste coming in daily is known, the life expectancy of landfills can be predicted.

Every day, around 6,500–7,000 tons of waste are dumped into Bantargebang landfill. This landfill has a total area of 110.3 Ha; 74.5% of it is the landfill area, while the rest 25.5% is for other infrastructure, namely access roads, offices, and leachate treatment plants [9]. The capacity of Bantargebang landfill is 49 million tons. Currently, it has accommodated 39 million tons of waste, meaning that the remaining capacity of Bantargebang landfill is only 10 million tons. If it is assumed that no less than 7,400 tons of waste are disposed of at Bantargebang landfill every day, it is estimated that this landfill will reach its maximum capacity within two following years [10]. Since the land is limited, waste management is extremely needed to reduce the amount of waste entering Bantargebang landfill. Thus, it is crucial to know the capacity of Bantargebang landfill based on the input of waste that comes in every day.

Landfill capacity is part of the waste management concept, which continues to develop over time. André and Cerdá [11] talk about landfill construction and capacity expansion. Meanwhile, Memon [12] discusses the concept of solid waste management. Waste management initially aims to reduce public health risks and later widens its focus to environmental aspects. In addition, resource conservation and resource recovery become critical factors for solid waste management based on the 3R approach (reduce, reuse, and recycle). According to Memon (2010), resource recovery is maximized at all stages of solid waste management. In his study, Memon also introduces a new terminology, i.e., integrated solid waste management, which streamlines all stages of waste management, which include source separation, collection, and transportation, transfer stations and material recovery, treatment and resource recovery, and final disposal. Another previous study by Ikhlayel, Higano, Yabar, and Mizunoya [13] examined the municipal solid waste management in the Middle East and North Africa region. The main problems associated with such waste management are poor collection rates, open dumping, and improper recycling, which cause environmental damages. The waste management system in developing countries, especially Bangladesh, has also been studied previously by Ahsan et al. [14].

Integrated solid waste management is a comprehensive waste prevention, recycling, composting, and disposal program. It also involves evaluating local needs and conditions and then selecting and combining the most appropriate waste management activities for those conditions. In Bangladesh, a sanitary landfill mechanism needs to be provided in existing ultimate disposal sites. Furthermore, government support should also be supplied for composting, which is the most profitable sector for managing large amounts of organic wastes in this country. All treatment activities for the waste must be well integrated.

While various studies related to waste management have been carried out previously, studies on waste management using models have not been widely done, especially in Indonesia. Related studies include those conducted by Park et al. [15] who examined the simulation methods of a system dynamic model for operations and planning of capacity expansion of activated-sludge wastewater treatment plants and by Surjandari et al. [16] who analyzed the dynamic model of waste management to reduce stacking loads. Another prior study by Mwanza and Phiri [17] proposed a design of a waste management model for Bulawayo City Council based on an integrated solid waste management system. Different from other studies, the objective of this study was to predict landfill capacity based on daily waste input using a system dynamic modeling. From the results, alternative policies are recommended as an implementation of the capacity management of Bantargebang landfill.

Prediction with a dynamic model makes it possible to simulate influential variables that can reduce waste generation. Simulations were carried out for quantities of organic waste and inorganic waste variables related to recycling and reusing. By simulating the amount of waste to be disposed of in the landfill, it is possible to know the limit of the landfill capacity. In addition to predicting landfill capacity in accommodating waste from population based on daily waste input using a system dynamic modeling, this study also aimed to provide alternative policies on landfill waste management.

## 2. Materials and Methods

This study applied a system dynamic approach by utilizing Vensim software to develop a waste management model for DKI Jakarta Province. System dynamics is used to analyze changes in behavior over time in a complex system [18]. Through system dynamics, changes in the system can be seen, especially to assess the effectiveness of policy plans made. The model shows a direct or indirect relationship and a causal relationship. It is an abstraction of reality and can be said to be complete if it can represent various aspects of the reality being studied [19].

The first stage in system dynamics is problem identification and emergence. Then, a computer model is built and outlined in a flow chart or stock and flow diagram (SFD), which describes the interrelationships of all variables. All data are input to SFD. Data can be in the form of stock (level), flow (rate), auxiliary, and constant. The next step is to simulate time or constant changes in variables. Model validation is carried out by comparing the simulation results with the characteristics of the empirical data using absolute mean error (AME).

Based on the steps explained above, both primary and secondary data collected in this study were input to the SFD. The primary source of data collection is in the form of interviews with expert staff at the office of Bantargebang landfill to find out the daily activities of the landfill. Meanwhile, the secondary data are in the form of documents obtained from Statistics Indonesia, the Environmental Services of DKI Jakarta, and the management office of Bantargebang landfill, as well as time-series data collected in 2008–2018. After the data were collected, the relationships between these variables were examined.

The next step was simulating time or constant changes in variables. Simulations were done on types of waste variables, namely organic waste and inorganic waste. Composting was chosen for organic waste, whereas recycling and reusing were for inorganic waste. Three model scenarios were constructed in this study, namely (1) organic waste processing by composting done by the community, with percentages of 25% and 50%; (2) inorganic waste processing by recycling and reusing, with percentages of 25% and 50% for both methods; and (3) combination of organic and inorganic waste processing to the total amount of waste. Finally, the last step was validating the models by comparing the simulation results with the characteristics of the empirical data using absolute mean error (AME).

## 3. Results

The population of Jakarta is increasing over the years with varying population growth. Along with the increase in population, the amount of waste generated by urban activities continues to rise, and the types of waste produced are also increasingly diverse. Based on the Decree of the Head of the Environmental Services of DKI Jakarta Provincial Government Number 699 of 2017 on Waste Generation, Composition, and Characteristics at Bantargebang landfill, 0.6091 kg to 0.75 kg of waste is produced per person each day [20]. This amount is greater than that of Ghana, which is 0.47 kg of waste/person/day [21].

### 3.1. Waste Management

**Figure 1:**
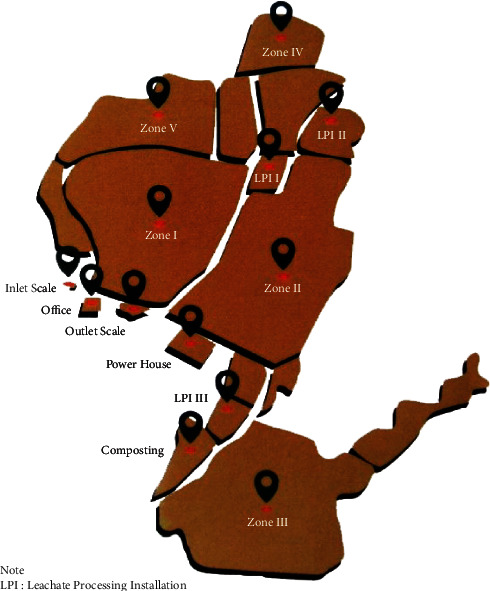
Map of Bantargebang landfill. Source: Integrated Waste Management Unit, the Environmental Services of DKI Jakarta Provincial Government, 2018 [9].

Waste originating from Jakarta is disposed of at Bantargebang landfill, which applies a sanitary landfill system.  Figure 1 shows the area and designation of Bantargebang landfill as a sanitary landfill.

**Figure 2:**
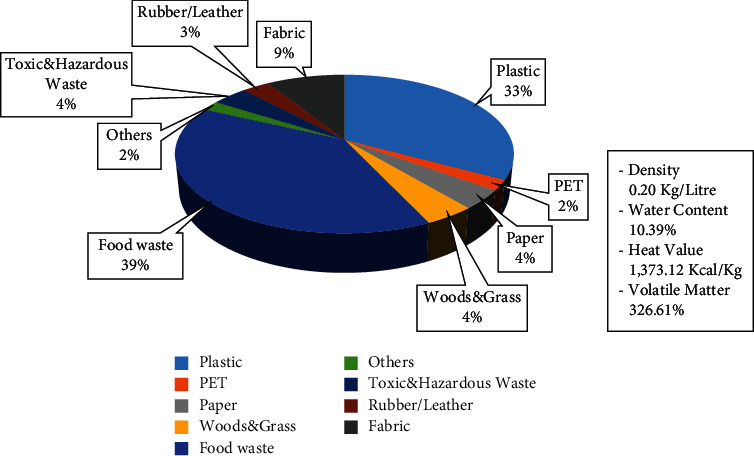
Composition and characteristics of waste in Bantargebang landfill.

Bantargebang landfill has a total area of 110.3 hectares, of which 81.91 hectares are divided into five zones and two leachate processing installations (LPIs). Each zone has a different function; Zone I and Zone II are covering landfill and waste-to-energy (WTE) plant, Zone III is covering landfill and pipe tidying plant, Zone IV is WTE plant, and Zone V is landfill mining and WTE plants [22]. At present, the height of waste in Bantargebang landfill has reached 20–30 meters. The LPI system uses two methods, namely the conventional method of aeration and coagulation (around 200 m^3^/day) and the advanced oxidation process with a combination of ozone, UV light, and hydrogen peroxide (65–70 m^3^/day) [9]. The composition and characteristics of waste entering Bantargebang landfill are shown in Figure 2.

Based on interviews with experts at Bantargebang landfill, it is known that waste management in Jakarta begins with household waste collection to the urban waste collection points by the community in the neighborhoods. Then, the waste is transported by trucks once a day to Bantargebang landfill. Around 1,200 trucks enter Bantargebang landfill daily, transporting approximately 6,000–7,000 tons of waste. Every vehicle that comes into Bantargebang landfill is recorded, validated, and weighed using a computer program. The demolition of waste from trucks to the point of disposal is carried out in a relay using heavy equipment. Composing is carried out for organic waste.

The waste is then flattened and compacted with heavy equipment. The daily cover is 20 cm thick. If the height of the waste reaches five meters, the landfill cover needed is 30 cm. After that, further processes are carried out for existing piles of garbage in the landfill, including terracing/countering landfills, powerhouses, or LPIs.

For composting process, the received organic waste is regularly mixed with heavy equipment for up to 30 days. Separation of nonorganic materials such as woods, stones, textiles, and plastics continues until the compost material turns to fine powder. Then, the powder is processed into granules. The next process is drying and packaging. The current compost production capacity is 20 tons/day [9]. The results of this compost are not commercialized, but distributed to surrounding communities and used for agriculture and plantations in Bantargebang landfill area.

Inorganic waste in Bantargebang landfill can be taken by scavengers. Approximately 10,000 scavengers become members of the association and are given social security. However, only about 20% of the total amount of plastic waste in this landfill can be taken out by scavengers since the location is difficult to reach.

### 3.2. System Dynamic Simulation

**Figure 3:**
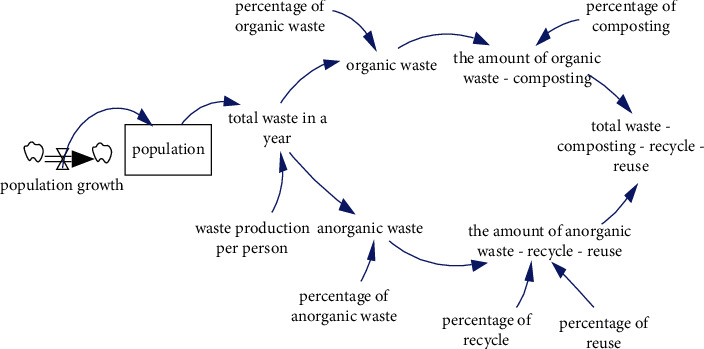
SFD of organic and inorganic waste processing with total accumulation of waste.

To minimize the amount of waste coming into Bantargebang landfill, simulations of waste processing were carried out for organic waste (composting) and inorganic (recycling and reusing) by assuming various waste processing scenarios. The stock and flow diagram (SFD) is presented in Figure 3.

The coefficients used in the simulations are population size, waste production, and waste composition. Based on data from the Statistics Indonesia regarding the population of DKI Jakarta Province in 2010–2017, the population growth in this province is around 1.05% [23, 24], and waste produced per person per day is 0.75 kg [20]. The type of waste is divided into two major groups, namely organic and inorganic wastes. Organic waste mainly consists of leftovers, grass, and wood, while inorganic waste includes paper, PET, plastic, fabric, rubber/leather, metal, glass, and hazardous toxic material. The composition of the waste is 59.01% organic and 40.98% inorganic [25]. The total percentage is not 100% because they are round down.

### 3.3. Simulation Results

The overall simulation results based on the scenarios are displayed in Table 1.

#### 3.3.1. Simulation of Population Growth and Waste Growth

**Figure 4:**
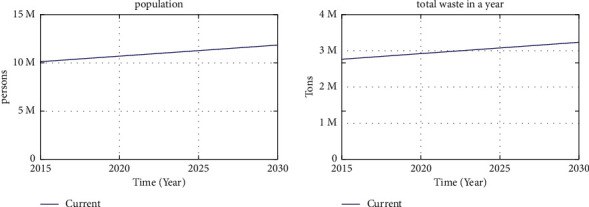
Simulation results of the total population of Jakarta and the amount of waste until 2030.

If nothing is done, the population of Jakarta will continue to grow to reach approximately 11,855,685 people by 2030, with the amount of waste generated being 3,251,330 tons (Figure 4).

As shown in Table 1 above, waste production increases over the years in line with the population growth. If no measures are taken from 2019 to 2030, the total amount of waste in Jakarta will reach 36,861,652.75 tons. Meanwhile, the remaining capacity of Bantargebang landfill is 10,000,000 tons. This means that Bantargebang landfill can only accommodate waste for three more years after 2019, assuming that all waste is accumulated in this landfill.

Based on the simulation results, the model was validated using the AME method. Validation was done by comparing the average empirical data with the average simulation model results. The results of the model validation are presented in Table 2.

The AME for the population is 0.18%. The model can be declared as valid since the acceptable deviation limit is 5–10% [26].

#### 3.3.2. Simulation Results with Various Scenarios

Based on three simulation scenarios, segregation at household level with 4 combinations can be assumed, namely

- 25% composted, 25% recycled, and 25% reused
- 50% composted, 25% recycled, and 25% reused
- 25% composted, 50% recycled, and 50% reused
- 50% composted, 50% recycled, and 50% reused

**Figure 5:**
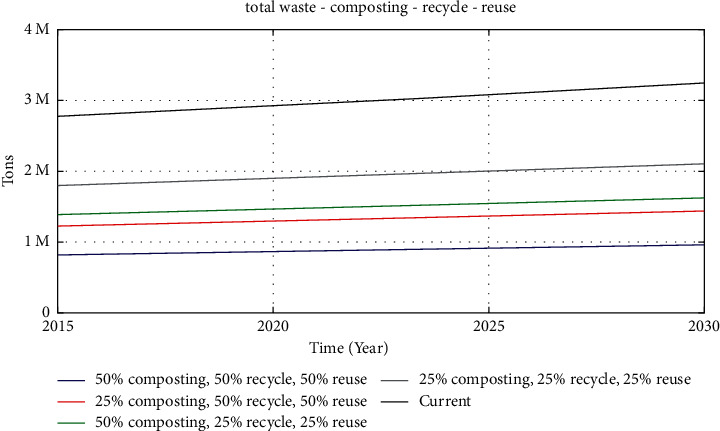
Simulation results with various scenarios of composting, recycling, and reusing.

These assumptions are based on the predictions that the waste going to Bantargebang landfill will be minimized. The simulation results are shown in Figure 5.

Based on the simulation results in Table 1, the waste management scenario of 25% composted, 25% recycled, and 25% reused can reduce 35.25% of the total amount of waste. With this scenario, Bantargebang landfill can only accommodate waste until 2023 before reaching its maximum capacity of 10,000,000 tons. Meanwhile, for the waste management scenario of 50% composted, 25% recycled, and 25% reused, the percentage of waste reduction can reach 50%. In this regard, Bantargebang landfill can be used up to 2025 (see Table 1).

As for the waste management scenario of 25% composted, 50% recycled, and 50% reused, the percentage of waste reduction can reach 56% and Bantargebang landfill can be used until 2026 (see Table 1). This is only slightly different from the previous simulation result (50% composted, 25% recycled, and 25% reused), meaning that the increase in the percentages of the recycle and reuse processes does not provide a significant change.

The last simulation is an optimistic condition, with 50% composted, 50% recycled, and 50% reused waste. Figure 5 shows that this simulation leads to the most significant reduction, with a percentage of 70.5%. With this condition, Bantargebang landfill can accommodate waste up to 2030 (see Table 1).

## 4. Discussion

Waste management in Bantargebang landfill is in line with a study conducted by Arthika et al. [27] who stated that the design of a sanitary landfill can be produced by estimating the population size and the total solid waste produced, as well as by providing leachate management systems and end systems for a period of 20 years. In addition, Skenderovic et al. [28] argued that the concept of waste management refers to the activities of collecting, transporting, sorting, recycling, disposing, tracking, and monitoring waste. The biggest problem arises in the collection of waste for recycling, e.g., waste sorting, since some parts of the process must be performed manually, thus increasing the costs involved. All waste management activities must be well integrated [29]. Regarding this matter, Krook and Eklund [30] developed a monitoring method that facilitates continual improvements in the sorting of waste at recycling centers.

Based on several simulations proposed previously, waste reduction by composting produces a significant result, compared with the other waste management methods, as the total amount of organic waste is greater than that of inorganic waste. According to Yang et al. [31], landfilled MSW of developing countries has high organic content ranging from 75.00% to 97.15% and the amount of food waste increases over time. On the other hand, the organic contents in landfills of developed regions are relatively small. In Indonesia, the percentage of organic waste only reaches 59.01% [25].

Waste processing done by each household in the community is proven to be able to significantly reduce the accumulation of waste. This must be done since the remaining capacity of Bantargebang landfill is extremely limited. Furthermore, the regional regulation also controls waste collection, which becomes the responsibility of waste management at the community level. People who collect household waste to the 3R waste processing plants must be fostered and supervised by the local government. Waste residues generated at the waste collection points are transported by the local government to final disposal sites or landfills at least twice a week. Additionally, the local government is required to provide a waste processing plant as regulated in the Guidelines for Preparation of Detailed Spatial Plan and Regency/City Zoning Regulations, which also specify transportation of waste. With regard to waste management, the regional regulation stipulates waste management to be available in villages, subdistricts, and other areas. Waste processing in the 3R temporary waste management sites at the village level should at least facilitate composting activities as an effort of the community to reduce waste generation. The compost produced at the 3R waste processing plant is used by the local government for maintenance, while the composting activities can be carried out in collaboration with business actors, business entities in the sanitation sector, and/or other local governments.

Another problem in waste management is the inadequate land available, leading to the incredibly limited individual waste conversion efforts. In rural areas, for example, waste cannot be professionally managed by the community itself. As an alternative, people in villages convert their wastes into compost or natural fertilizer for crops, dump them on the ground, or burn them.

Regarding the life expectancy of landfill, Akyen et al. [32] conducted a study on the estimation of the sustainability of landfills in Ghana. The study used the future value of money equation to estimate the lifespan of the Aboso landfill in Tarkwa, Ghana, which can accommodate MSW until 2029. From 2030 onward, the Aboso landfill will have exceeded its capacity. Therefore, a new landfill site will have to be developed by the government to manage the waste in the future.

### 4.1. Recommendations for Alternative Policies as an Implementation of the Capacity Management of Bantargebang Landfill

As an implementation of the management of Bantargebang landfill, which will reach its maximum capacity in few years, DKI Jakarta Provincial Government and the community need to do the following:

- Waste segregation by separating organic waste and inorganic waste must be done in every household. The marking of the type of waste is needed so that all family members can play a role in sorting household waste.
- After being sorted, the waste can be disposed of in temporary waste disposal sites, in which organic and inorganic wastes are also separated. To make it easier for the community to sort waste, the bins can also be distinguished by colors and marked.
- Separation is also carried out on the means of transportation from temporary disposal sites to final disposal sites or landfills.
- It is strongly recommended that organic and inorganic waste management must be separated. For example, local governments can manage organic waste, whereas inorganic waste management is done by recycling activists. Organic waste management can also involve or cooperate with private parties or other agencies engaged in the agricultural sector and in the provision of fertilizers.

## 5. Conclusion

Simulations with a system dynamic model can be used to predict how long Bantargebang landfill will be able to accommodate waste until it reaches full capacity. If waste management is not carried out properly, the amount of garbage entering this limited area will increase every day. Thus, waste processing must be done to reduce the amount of waste that comes into Bantargebang landfill.

If no measures are taken, the total waste production from 2019 to 2030 will reach 36,861,653 tons. According to data, Bantargebang landfill can only accommodate waste for around four years from 2018, namely until 2022, assuming that all waste from the surrounding communities goes to this landfill. To be able to use Bantargebang landfill up to 2030, the waste production must be reduced by 50% for organic waste and 50% for inorganic waste.

Simulations were carried out on waste reduction by composting for organic waste and by recycling and reusing for inorganic waste. Based on the results, composting is proven to reduce greater amount of waste than the other methods by the same percentages. Waste sorting is highly recommended to be done by the community in every household. The community can play an active role in reducing waste by composting organic waste and recycling or reusing inorganic waste. Furthermore, regulations should be made that can give punishment to those refusing to carry out segregation. Reliable infrastructure for waste management needs to be facilitated and counseling/training/outreach on waste sorting to the community must also be provided at the district level.

## Figures and Tables

**Table 1:** Simulation results with various scenarios.

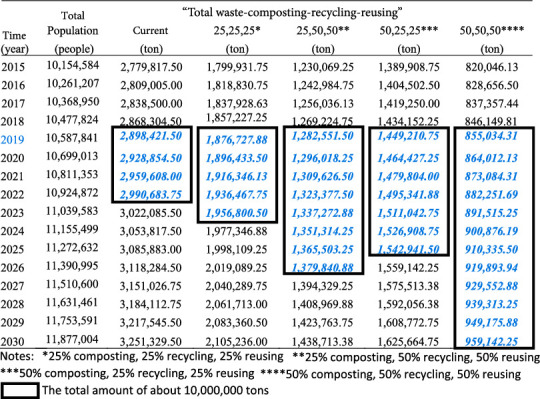

**Table 2:** AME of population.

Variable	Average		AME (%)
	Empirical	Simulation	
Population	10,350,338	10,368,950	0.18

## Data Availability

Data are available upon request from the corresponding author by interviews with expert staff at the office of Bantargebang landfill and from the Statistics Indonesia of DKI Jakarta Province.
